# The immune mechanisms of acute exacerbations of idiopathic pulmonary fibrosis

**DOI:** 10.3389/fimmu.2024.1450688

**Published:** 2024-12-16

**Authors:** Tao Chen, Wei Sun, Zuo-jun Xu

**Affiliations:** ^1^ Department of Respiratory and Critical Medicine, Peking Union Medical College Hospital, Chinese Academy of Medical Sciences and Peking Union Medical College, Beijing, China; ^2^ Department of Respiratory and Critical Medicine, The second hospital of Tianjin Medical University, Tianjin, China

**Keywords:** acute exacerbations of idiopathic pulmonary fibrosis (AE-IPF), immune mechanism, macrophages, Th17, acute lung injury

## Abstract

Acute exacerbations of idiopathic pulmonary fibrosis (AE-IPF) are the leading cause of mortality among patients with IPF. There is still a lack of effective treatments for AE-IPF, resulting in a hospitalization mortality rate as high as 70%–80%. To reveal the complicated mechanism of AE-IPF, more attention has been paid to its disturbed immune environment, as patients with IPF exhibit deficiencies in pathogen defense due to local immune dysregulation. During the development of AE-IPF, the classical stimulatory signals in adaptive immunity are inhibited, while the nonclassical immune reactions (Th17) are activated, attracting numerous neutrophils and monocytes to lung tissues. However, there is limited information about the specific changes in the immune response of AE-IPF. We summarized the immune mechanisms of AE-IPF in this review.

## A brief introduction

Idiopathic pulmonary fibrosis (IPF) is an interstitial lung disease (ILD) characterized by progressive pulmonary fibrosis and a continuous decline of pulmonary function. The median survival time of IPF is 2–3 years (1). Acute exacerbation of IPF (AE-IPF) is an acute, clinically significant respiratory deterioration characterized by evidence of new widespread alveolar abnormality (2). The etiology of AE-IPF includes infection, micro-aspiration, surgical procedures, and mechanical stretch (3, 4). The prognosis of AE-IPF induced by these causes is quite similar (5–7). The reported hospitalization mortality rate of AE-IPF is 70%–80%. However, the underlying mechanisms of AE-IPF are poorly understood. Exploring the underlying immune mechanisms is helpful to understand clinical characteristics and develop effective treatments.

Studies have found that patients with pulmonary fibrosis had a higher bacteria load (6, 8) and virus (9). Disease progression of IPF was associated with the presence of specific pathogens (4, 6), including *Staphylococcus* and *Streptococcus* genera (10, 11). However, a randomized clinical trial demonstrated that antibiotic treatment failed to improve the prognosis of IPF compared with placebo (12). These results suggest that patients with IPF are more susceptible to infections, and the microbiota of the respiratory tract is changed in IPF due to the changes in host–microbiome interactions. A recent study investigated spatial gene expression in different regions of fibrotic lung tissue, and they found that the lung tissues adjacent to fibrotic foci showed dysregulated innate immunity and inflammatory signatures. Specifically, loss of NF-κB inhibitor zeta might impair homeostatic responses of alveolar epithelial cells (AECs) to environmental stress by dysregulating the TGFβ/IL-6 signaling axis (13). An *in vivo* experiment illustrated that mice with established pulmonary fibrosis had a weaker ability to defend against bacterial infection, characterized by decreased levels of alveolar macrophages and aggravated cytokine/chemokine responses (14).

Except for the infection, other factors, inducing the activation of immune systems, were also identified as the etiologies of AE-IPF, including aspiration, air pollution exposures, surgery operation, trauma, and mechanical injuries (2). In a PM-2.5 particle-induced AE-IPF mouse model, researchers treated mice with PM2.5 after establishing pulmonary fibrosis with bleomycin. PM2.5 treatment induced recruitment of monocytes to the lung, and these monocytes showed enhanced expression of proinflammatory markers (15). As for the mechanical injury in AE-IPF, a previous study compared patients with AE-IPF with other patients with acute respiratory distress syndrome (ARDS). They found that patients with AE-IPF presented higher baseline esophageal pressure swing, respiratory rate, and dynamic mechanical power compared with ARDS patients. Moreover, the non-invasive ventilation reduced the esophageal pressure swing and respiratory rate, but did not change the dynamic mechanical power and dynamic transpulmonary pressure in patients with AE-IPF (16). These results indicated that patients with AE-IPF were more susceptible to the respiratory mechanical injuries. Under hypoxic conditions, patients with AE-IPF need a stronger respiratory driving force for respiration, which might cause lung tissue injury and elicit acute inflammation. The above mechanisms might partly explain the acute exacerbations without a known cause in patients of IPF.

Actually, any factors, which can induce the systemic inflammation, could initiate the immune reactions of acute lung injury. The mature fibrotic lung tissues, presenting as usual interstitial pneumonia on HRCT, can be treated as an immune organ, as the macrophages take more than 50% of cellular components. Therefore, a small insult will cause explosive production of inflammatory cytokines and eventually elicit acute lung injuries. As a result of it, some conditions, such as a common episode of cold, trauma, or a small surgery operation, could cause ARDS in patients with IPF. To date, many data have indicated that the imbalance of immune function might be the fundamental mechanism of AE-IPF (17). We will summarize the immune mechanisms of AE-IPF in the present review.

## M1 and M2 in AE-IPF

Two nomenclature systems have been used to annotate macrophages in previous studies. One system is derived from the function of macrophages, grouping them into M1 and M2 categories. The other system is based on the trajectories of differentiation, such as monocyte-derived macrophages (Mo-M) and tissue-resident alveolar macrophages (TR-AM). Previous studies have used different marker proteins to identify Mo-M, including CD11b^+^CD64^+^F4/80^low^Siglec F^low^ (18), and the inconsistency makes it difficult to compare the results. M1 and M2 are traditionally defined phenotypes of macrophages. M1 macrophages secrete pro-inflammatory cytokines to defend against pathogens and clear cell debris. M2 macrophages secrete immune-regulatory cytokines to suppress acute inflammation and promote fibrosis (19–21). M1 is induced by LPS, IFN-γ, and GM-CSF. The surface markers of M1 include CD80, TLR4, MHCII, and CD86. The inflammatory factors secreted by M1 macrophages include NO, IL-1β, IL-12, IL-23, CCL2, and TNFα (22–24). In contrast, M2 macrophages are induced by IL-4, IL-13, TGFβ, and IL-10 (25). The surface markers of M2 include CD206, CD163, CD209, FIZZ1, and Ym1/2. M2 macrophages produce IL-10, TGF-beta, CCL1, CXCL13, and VEGF to shape the fibrogenic environment (26–29). In pulmonary fibrosis, both alveolar macrophages (CD11b ^low^ CD11c^++^ CD169^+^) and interstitial macrophages (CD11b^+^ CD11c^low^ CD169^−^) can differentiate into M1 and M2; moreover, the chemotactic monocytes are also the precursors of M1 and M2 (30, 31).

M2 macrophages are recognized as a fibrogenic phenotype. It is the main phenotype of macrophage in IPF. However, not just M2 resides in the lung tissues of IPF. Some lung tissues of IPF might predominate with M1, which shape the immune environment with acute inflammation in the areas with alveolar epithelial injuries. M1 and M2 simultaneously exist in the lung tissues of IPF, which is determined by the immune microenvironment (32). M1 and M2 can transform into each other under specific conditions (33, 34). For example, when patients with IPF have acute infections, the M2-dominant circumstances will transfer to an M1-dominant environment, which induce M2 to differentiate into M1 with increased inflammatory cytokines of TNFα and IL-6 (35). Therefore, it would be better to define a phenotype of immune cells while describing the immune environment at the same time.

## Macrophages are deficient in defending against pathogens in AE-IPF

**Figure 1:**
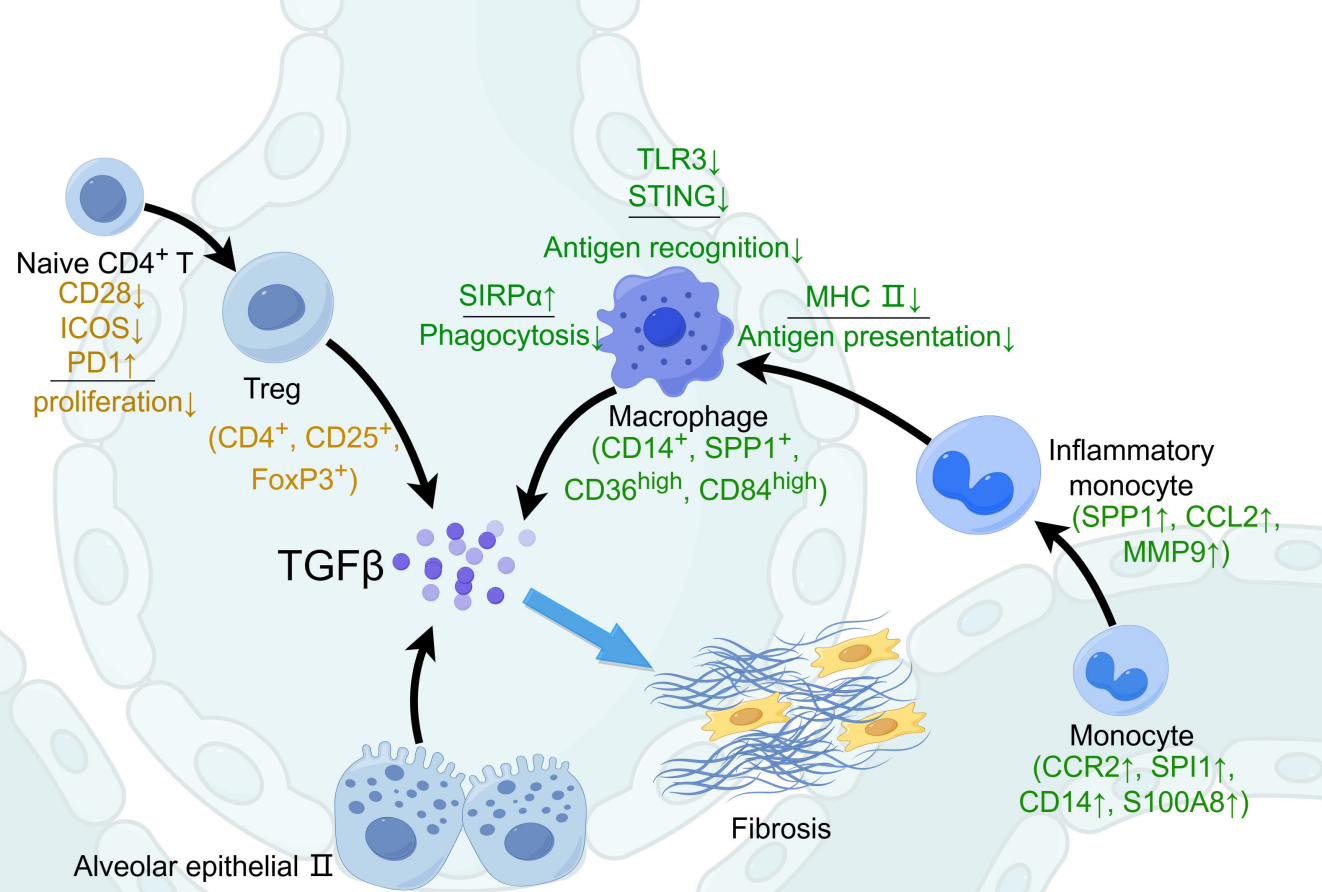
The mechanism of idiopathic pulmonary fibrosis. In the niche of pulmonary fibrosis, macrophages are deficient in antigen recognition, phagocytosis, and antigen presentation. The surface markers of the fibrogenic macrophages are CD14^+^, SPP1^+^, CD36^high^, and CD84^high^. In such circumstances, Naïve CD4^+^ T lymphocytes are differentiated into Treg cells. TGFβ are mostly secreted by Treg, fibrogenic macrophages, and AECII. This figure was created by Figdraw (ID: UWSAI35444; www.figdraw.com). CCR2, C-C motif chemokine receptor-2; CCL2, C-C motif chemokine ligand-2; ICOS, inducible T-cell costimulatory; MHCII, major histocompatibility complex II; MMP9, matrix metalloproteinase-9; PD-1, programmed cell death protein-1; SIRPα, signal-regulatory protein-α; SPP1, osteopontin; STING, stimulator of interferon genes; TLR3, Toll-like receptor 3.

In IPF, the weakened ability to defend against infections could be partly explained by the decreased ability of alveolar macrophages in antigen recognition, phagocytosis, and antigen presentation (**Figure 1**). First, the Toll-like receptors are the most important antigen-recognition molecules on the surface of the macrophages. Studies have reported that about half of patients with IPF had defective TLR3 function, and losing the function of TLR was associated with an increased risk of mortality and an accelerated decline in FVC in IPF (36, 37). Specifically, a mutation in TLR3 attenuated the response of macrophages to bacteria and viruses and induced the infection-related acute exacerbations of IPF (37). Furthermore, macrophages from patients with IPF also exhibited decreased ability to defend against the virus infection, due to the deficiency of stimulator of interferon genes (STING) (38), an essential recognition molecule of virus. A spatial transcriptome study illustrated that type I IFN-regulated gene expression was suppressed in IPF lung tissues (13). Moreover, HSV1 infection in addition to bleomycin can successfully establish a murine model of acute exacerbations of IPF (39).

Second, studies have demonstrated the deficiency in efferocytosis of alveolar macrophages in patients with IPF (40), which was partially explained by the disturbance of mitochondrial homeostasis (41). Moreover, tumor-associated macrophages (TAMs), which are M2 phenotype, also have decreased phagocytic capacity (42). Alveolar macrophages from patients recovered from primary pneumonia exhibited poor phagocytic capacity for several weeks in a SIRPα-dependent way, and blockade of SIRPα restored phagocytosis (43). The immune environment of post-pneumonia is similar to that of chronic fibrosis. SIRPα is also increasingly expressed in macrophages in pulmonary and hepatic fibrosis (44, 45). Therefore, macrophages from IPF also displayed a reduction in phagocytosis.

Third, M2 in the fibrotic foci expressed a lower level of MHC II (20, 46). Decreased MHC II weakens the capacity of antigen presentation. Traditionally, M2 is characterized by the function of inhibiting inflammation; therefore, it has weaker antigen presentation capacity (47). A previous study illustrated that Th2 inflammation increased the bacteria burden in a mouse model of *Klebsiella pneumoniae* infection, which was characterized by reduced levels of alveolar macrophages and increased immune cell infiltration (48). Taken together, macrophages from IPF exhibit deficiency in antigen recognition, phagocytosis, and antigen presentation, resulting in a higher load of pathogens and the delay of pathogen clearance.

## The canonical adaptive immune responses are inhibited in AE-IPF

**Figure 2:**
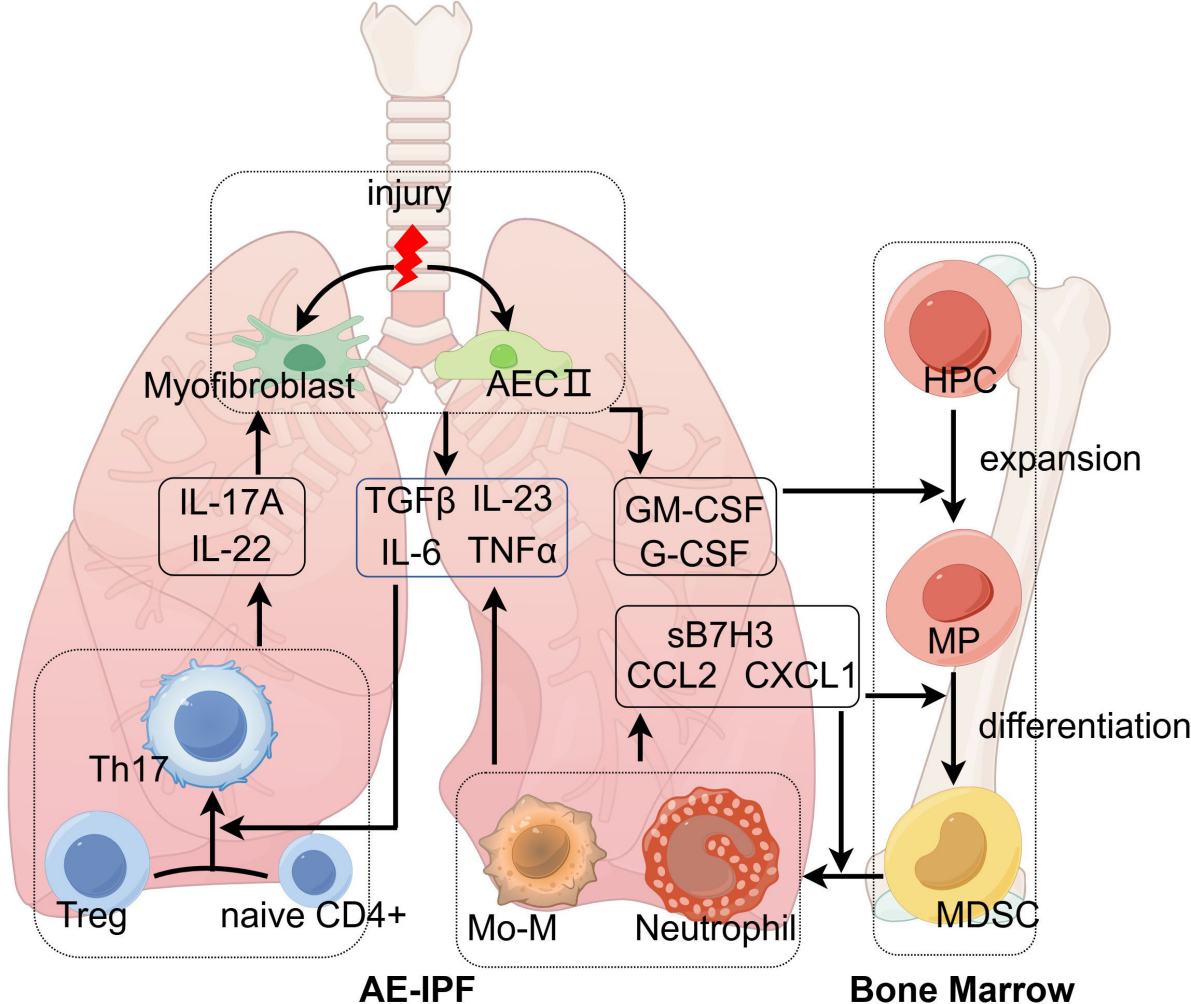
The mechanism of acute exacerbations of idiopathic pulmonary fibrosis. Environmental stress induces type II alveolar epithelial cells (AECII) and myofibroblasts to release GM-CSF and G-CSF, which are essential for the differentiation of hematopoietic progenitor cells (HPCs). With the help of chemokines, neutrophils and monocyte-derived macrophages (Mo-M) traffic to injured lung tissues and secrete acute inflammatory cytokines (IL-6, IL-23, and TNFα), which transform innate lymphocytes and Treg into Th17. Th17 secretes IL-17A and IL-22 acting on AECII and myofibroblasts to promote releasing GM-CSF and G-CSF. The positive feedback loop eventually leads to acute exacerbations of IPF. This figure was created by Figdraw (ID: AWRWUcba31; www.figdraw.com). AECII, type II alveolar epithelial cell; CCL2, C-C motif chemokine ligand-2; CXCL1, C-X-C motif chemokine ligand-1; GM-CSF, granulocyte-macrophage colony-stimulating factor; G-CSF, granulocyte colony-stimulating factor; HPC, hematopoietic progenitor cells; IL, interleukin; Mo-M, monocyte-derived macrophages; MDSC, myeloid-derived suppressor cells; MP, myeloid precursors; TGFβ, transforming growth factor-β; Th17, T helper cell 17; TNFα, tumor necrosis factor-α; Treg, regulatory T cells.

For patients with IPF, adaptive immunity is suppressed because of insufficient stimulus from innate immunity. The lack of costimulatory signals between APC and CD4+ T lymphocytes is another important part of the immune mechanism in AE-IPF (**Figure 2**). Recently, some studies have illustrated that a higher level of lymphocytes either in blood or in bronchoalveolar lavage fluid (BALF) was associated with better prognosis in patients with ILD. Patients with IPF had significantly better prognosis if they had a lower neutrophil-to-lymphocyte ratio (49). A recent study performed single-cell RNA sequencing (scRNA-seq) on PBMC from 25 patients with IPF, and it found decreased lymphocytes in IPF versus healthy controls, and progressive versus stable IPF. However, Tregs were increased in progressive IPF and associated with decreased survival (50). Furthermore, Joseph and colleagues found that patients with UIP patterns or extensive fibrosis lesions were rare to have an increased BAL lymphocyte proportion. An increased BAL lymphocyte proportion was associated with a decreased probability of disease progression in patients with non-extensive fibrosis or a non-UIP pattern (51). These results illustrated that a higher lymphocyte load in lung tissues was associated with a better prognosis in ILD. There are two possible explanations for decreased lymphocytes. The one is that, in the lung tissues of UIP pattern or extensive fibrosis, the inhibited innate immune system fails to stimulate adaptive immune reaction (52). The other is that in these lung tissues, the function of lymphocytes is also inhibited by the fibrogenic niche. These two factors result in anergy and exhaustion of CD4+ T lymphocytes.

CD40L, ICOS, and CD28 are vital costimulatory molecules in T lymphocytes. Patients with IPF have a lower expression of CD28 and CD40L and a higher PD-1 expression compared with healthy controls (53, 54). Studies have demonstrated that the higher expression of ICOS on peripheral CD4 T cells predicted favorable survival outcomes for patients with IPF (55). In another study, authors enrolled two independent cohorts with patients with IPF, used different microarray platforms, and found that decreased expression of the genes of “The costimulatory signal during T cell activation” (CD28, ICOS, LCK, and ITK) was associated with shorter survival time in both cohorts (56). Downregulation of ICOS and CD28 was also associated with worsening pulmonary function (57). Another study analyzed the phenotypes of T and B lymphocytes in lung-draining lymph nodes (LLNs) of patients with ILD, and they found that patients with ILD with a history of acute exacerbation had lower expression of ICOS. Furthermore, a low-CD25 subset of Tregs was highly enriched in the LLN of ILD. Low CD25 expression is a phenotype of T follicular regulatory cells (Tfr), which can inhibit interactions between T follicular helper cells and B cells or prevent autoantibody production (58). Moreover, T lymphocytes in the LLN of patients with ILD had a significantly reduced expression of CCR7, indicating that these cells were poised to leave the LLN and migrate back to the tissue (59). Taken together, the above results suggest that the anergy of adaptive immunity is associated with a worse prognosis of IPF.

Schott and colleagues explored the association between lymphocyte dysfunction and the progress of ILD. They used the publicly available data of IPF to perform a consensus-weighted gene correlation network analysis and found that reduced expression of lymphocyte activation genes was identified in patients with IPF compared with control subjects, which was highly correlated with decline of DL_CO_ and survival rates (53). The inhibited function of lymphocytes is consistent with the clinical situation that corticosteroids are less effective in IPF compared with other ILDs, as the main effect of corticosteroids is suppressing the function of lymphocytes. Furthermore, they also illustrated that Th1 is inhibited in the sustained fibrogenic immune niche (60). Th1-related cytokines, IL-12 and IFN-γ, ameliorate fibrosis by inducing the production of matrix metalloproteinases (61, 62). Researchers have demonstrated that patients with IPF had decreased levels of IL-12 and IFN-γ in plasma and BALF (63). As we know, Th1 is vital for fighting against pathogens. As the Th1 reaction is inhibited, the nonclassical reaction of Th17 and Treg is activated when patients with IPF have acute infections or injuries (64).

## The specific phenotype of macrophages in AE-IPF

With the development of single-cell omics, researchers have found a special phenotype of M2 macrophage (65), the scar-associated macrophages (SAM), which are located in the lesions undergoing fibrotic processes. SAM expresses some unique markers, which are different from the traditional M2. Specifically, SAM highly expresses SPP1, MERTK, CHI3L1, FABP5, CD63, CD9, CD36, CD84, and TREM2 (65–69), in which CD14^+^, SPP1^+^, CD36^high^, and CD84^high^ can be used as the marker proteins. These markers are derived from the studies performing scRNA-seq with the lung tissues of IPF (**Table 1**). Kentaro and colleagues conducted a mass cytometry analysis of immune cell subsets in BALF from patients with IPF and found that CD14^+^ CD36^hi^ CD84^hi^ CCR2^–^ monocytes were increased in patients with IPF and the progressive phenotype of ILD (69). In another study, CD36^hi^ CD84^hi^ macrophages were also identified in lung tissues of IPF by a combination of scRNA-seq, cytometry by time of flight (CyTOF), and flow cytometry (65). In patients with fibrotic hypersensitivity pneumonitis and systemic sclerosis-associated ILD, SPP^hi^ macrophages were also associated with lung tissue fibrosis (70, 71). Moreover, previous studies have demonstrated the profibrotic potential of SAM across multiple tissues, including the liver, kidney, and heart (72). All these studies demonstrated that SPP1 was significantly increased in SAM. The protein product of SPP1 is osteopontin, which has two main functions. On the one hand, osteopontin is an integral part of the mineralized matrix as a non-collagenous bone protein. On the other hand, it is a cytokine involved in type I immunity by enhancing the production of IFN-γ and IL-12 (73). Studies have found that circulatory osteopontin was a marker of fibrosis progression in patients with IPF (74), and decreasing osteopontin expression attenuated pulmonary fibrosis in mouse models (75). The receptor of osteopontin is CD44, expressed on fibroblasts and epithelial cells, which activates the downstream signal of PI3K-Akt (76).

**Table 1:** The markers for monocyte or macrophage specific to pulmonary fibrosis.

Cell type	Markers	Technology	Cell source	Patients
Macrophage (66)	CD9 *, TREM2 SPP1, GPNMB, FABP5	scRNA-seq	Lung tissues	10 SSc-ILD 56 IPF
Macrophage (141)	SPP1, CHI3L1, LIPA	scRNA-seq	Lung tissues	32 IPF 28 HC
Macrophage (65)	**CD36**, **CD84**, SPP1, GPC4, **TREM2**, **CD9**, APOE, LILRB4, **CD276**, SLAMF7	scRNA-seq	Lung tissues	32 IPF, 29 HC
Macrophage (68)	CHI3L1, SPP1	scRNA-seq	Lung tissue	4 IPF, 3 CTD-ILD, 1 CHP, 8 HC
Macrophage (67)	SPP1, **MERTK**	scRNA-seq	Lung tissues	3 IPF 3 HC
Macrophage (142)	SPP1	scRNA-seq	Lung tissues	8 IPF, 8 SSc-ILD, 5 HC
Monocyte (69)	**CD14^+^ CD36^hi^ CD84^hi^ CCR2^–^**	Mass cytometry	BALF	8 IPF, 13 CTD-ILD, 10 SA
Monocyte (143)	**CD14+ CD64+**	Flow cytometry	PBMC	37 IPF, 28 HC
Monocyte (82)	**CD14+**CCL3^hi^ CCL4^hi^ S100A^hi^	scRNA-seq	PBMC	46 IPF, 14 FHP 5 non-FHP, 31 HC
Macrophage (82)	SPP1, CCL2, MMP9, **CD68**, **MERTK**, CCL18	scRNA-seq	BALF	10 IPF 6 FHP 4 non-FHP
Macrophage (144)	SPP1, LPL, CHIT1	scRNA-seq	Lung tissues	IPF, SSc-ILD, HP
Macrophage (70)	SPP1^hi^	scRNA-seq	PBMC and BALF	43 FHP, 63 IPF, 4 non-FHP, 36 HC
Macrophage (71)	SPP1, CCL2, **MERTK**	scRNA-seq	Lung tissue	8 SSc-ILD, 5 HC

*Bold indicates the surface protein on cell membrane.

Single-cell omics have characterized the immune cellular diversity of human lung tissues with pulmonary fibrosis. Some limitations deserve mention. First, most studies perform the scRNA-seq using lung tissues. However, the standard sequencing procedure captures about 10,000 cells once, usually separated from less than 1 cm^3^ of lung tissues. The experiment results are highly dependent on where you get the sample. For example, the lower and subpleural lung tissues had more contents of fibrosis and less inflammation compared with the upper and central lung tissues. Second, the RNA sequence cannot represent the features of protein expression, as there are many mechanisms of RNA modification affecting the expression of proteins. Third, the expressions of RNA are easily changed in the experiment process before the sequence. For example, the changes in temperature and procedures of cell separation might influence the results. Therefore, only a high-level consistency in the pre-sequence experimental procedure can ensure a reliable result. Last, there has been no analysis method to integrate the data from different experiments using different platforms.

Myeloid-derived suppressor cells (MDSCs) might be the precursor cells of SAM. Except for the expansion of traditional monocytes and polymorphonuclear cells, patients with IPF had a significant increase in MDSCs, which was inversely correlated with the pulmonary function of patients with IPF (52, 77, 78). MDSCs were able to activate resident lung fibroblast and/or myofibroblast through TGFβ production in a paracrine manner (79). Furthermore, MDSCs suppressed autologous T-cell function and promoted Treg differentiation by inhibiting the co-stimulatory T-cell signals (52). MDSCs are classified into two groups, monocytic MDSC (M-MDSC) and granulocytic MDSC (G-MDSC). In humans, the gate strategy of MDSC is CD33^+^CD11b^+^HLA-DR^−/lo^, among which CD14 and CD15 distinguish M-MDSC (CD14^+^CD15^low^) and G-MDSC (CD14^low^CD15^+^) (80). In the fibrotic lesions, most MDSCs differentiated into SAM with the help of IL-17A, GM-CSF, and TGFβ (66), which were similar to the process of differentiation from MDSCs to TAMs in tumors (81). Recently, Zhao and colleagues integrated single-cell RNA sequencing data of immune cells from peripheral blood and BALF. They found that circulating monocytes with high expression of CCR2, SPI1, CD14, and S100A8 were increased in patients with ILD, which were the main monocytes chemotactic to fibrotic lung tissues. Next, these monocytes differentiated to an intermediate phenotype of macrophages with increased expression of SPP1, CCL2, and MMP9. Finally, the mature SAM highly expressed CD68, MERTK, and CCL18 (82) in fibrotic lesions of IPF.

MDSC expansion is induced by various cytokines, such as GM-CSF, M-CSF, G-CSF, and VEGF, which are produced by injured lung tissues, tumors, or bone marrow stroma, especially when acute injuries occur in tissues with chronic inflammation. In patients with IPF, MDSCs are expanded and activated with the stimulation of GM-CSF/sB7H3 produced by injured lung tissues (83). B7H3 is a member of the B7 family that plays a critical role in the development of AE-IPF. Researchers have found that sB7H3 was increased in the BALF of progressive IPF compared with stable IPF. Mechanistically, sB7H3 initiated the alteration of the bone marrow environment caused by pulmonary fibrosis. This alteration in bone marrow environment was one of the key mechanisms for AE-IPF. A recent study found that mice that received bone marrow from BLM-pretreated donors showed significant exacerbation of fibrosis upon subsequent bleomycin treatment, while B7H3 was increased in bone marrow-derived antigen-presenting cells (CD45^+^CD11c^+^) (84). Generally, MDSCs are induced under chronic inflammation conditions and are essential to maintain the fibrotic immune niche in pulmonary fibrosis through secreting TGFβ and interacting with Treg/Th17.

The synergistic effects between acute injuries and fibrotic niche result in hyperinflammation in AE-IPF. When patients with IPF have acute infections or injuries, the macrophages and AECs have an explosive production of inflammatory cytokines and chemokines, which will induce the chemotaxis of monocytes and polymorphonuclear cells (85). It has been demonstrated that the synergism of TGFβ and pathogen receptors on macrophages promoted acute inflammation. Similar synergistic interactions have been demonstrated between IL-4 and TLR4 on macrophages. Researchers found that IL-4 pre-treated macrophages turned on a hyperinflammatory gene expression program upon TLR activation (86). In AE-IPF, this synergistic reaction might be through the activation of macrophage-inducible C-type lectin (Mincle), which is increasingly expressed on Mo-M of AE-IPF lung tissue. Mincle is involved in the process of defending against infection by promoting Th17 differentiation (87). Moreover, an intracellular receptor of foreign double-strained DNA in macrophages, named AIM2 inflammasome, was illustrated to promote the inflammation during AE-IPF induced by *Streptococcus pneumoniae*, which was regulated by glucose transporter 1 (GLUT1)-dependent glycolysis (88). Both Mincle and AIM2 promoted the secretion of inflammatory factors, such as IL-1β, TNFα, IL-18, and IL-6, and then increased the differentiation of Th17 with the help of TGFβ (89).

## Disturbed Treg/Th17 balance in AE-IPF

Both Th1 and Th2 are inhibited during AE-IPF (85). Instead, Treg and Th17 dominate the adaptive immune reaction in the development of AE-IPF. Treg has contrary functions in studies of pulmonary fibrosis (90). Young and colleagues analyzed adaptive immune cell phenotypes of lung-associated lymph nodes (LLN) from patients with ILD and healthy controls, and found that total Tregs and a CD25-low subset of Tregs were highly enriched in ILD LLN (59). Traditionally, Treg secretes IL-10 and TGFβ, which inhibit acute inflammation and promote fibrosis. Depletion of Treg in the early stage of BLM-induced pulmonary fibrosis leads to reduced fibrotic lesions (91). Another study induced the proliferation of Treg via IL-2 complex injection in mice, which worsened lung fibrosis after BLM injury (92). However, some studies found that Treg could reduce fibrosis when it was preventively transformed into a BLM-induced fibrotic model (90, 93). Treg was the most important antifibrotic effector cell of bone marrow−derived mesenchymal stromal cells in the BLM-induced mouse model (94). Another study, illustrating the antifibrotic potential of aryl hydrocarbon receptor, found that stimulation of aryl hydrocarbon receptor alleviated the BLM-induced pulmonary fibrosis in mice, which was concomitant with an increase in CD4+FoxP3+ Treg (95).

There is limited evidence for the role of Treg in acute exacerbations of IPF. One study found that Treg limits disease progression in bacteria-triggered fibrosis exacerbation (96). Researchers found an increase of Treg in AE-IPF mice, and depletion of Treg worsened infection-induced fibrosis, while IL-2 complex-induced Treg expansion with established lung fibrosis inhibited fibrosis exacerbation induced by pneumococcal infection. However, Treg can also transform to Th17 and secrete IL-17A with stimulation of IL-6 (97, 98). Researchers have found that CD4+FoxP3+ lymphocytes trans-differentiated into TH17 cells in a mouse model of autoimmune arthritis (97). A similar transition from Treg to Th17 was also illustrated in the asthma mouse model (98). From this perspective, Treg also has the potential to promote inflammation in AE-IPF. In the trajectories of T-cell differentiation, Treg and Th17 cells are relatively naïve compared to Th1 and Th2 cells, which diminishes their ability to interact with B cells and subsequently impairs antibody production. Furthermore, a significant disadvantage for patients with IPF is the reduction in memory T cells. A study has demonstrated that a higher proportion of memory T cells is associated with improved survival rates and better pulmonary function in patients with IPF (99). Collectively, these factors contribute to the compromised adaptive immune response in patients with AE-IPF.

IL-17 was increased in the plasma of patients with AE-IPF compared with patients with stable IPF (9). The ratio of Th17/Treg cells in lung tissues was higher in patients with AE compared with patients with stable fibrosis (100). In AE-IPF, IL-17 is significantly increased, which is secreted from regenerating epithelial cells and T lymphocytes by the stimulation of pathogens (39, 87, 101). IL-23, an essential inflammatory cytokine, was demonstrated to increase in BALF of patients with AE-IPF. In the murine model, knockout IL-23 reduced airway inflammation and fibrosis associated with decreased levels of interleukin-17A (102). In the AE-IPF mouse model with BLM and HSV1, IL-17A knockout alleviates acute lung injury by decreasing chemotaxis of neutrophils, and reducing levels of KC, IL-6, and TNFα in BALF (39). Similarly, in another AE-IPF mouse model with BLM and *Haemophilus influenzae*, IL-17 knockout mice with AE-IPF had quicker body weight recovery, milder pulmonary inflammation and fibrosis, and weaker neutrophil and eosinophil responses than wild-type mice with AE-IPF (103). Another IL-17 family member, IL-17B, is involved in the fibrogenic process induced by dysregulated lung microbiota. A study illustrated that commensal microbes produced outer membrane vesicles inducing IL-17B, which increased the expression of neutrophil-recruiting genes and Th17-cell-promoting genes and finally exacerbated lung inflammation and fibrosis (104). Th17 was also increased in the peripheral blood of patients with CTD-ILD, and this study demonstrated that Th17 reaction might contribute to the development of CTD-ILD (105). In conclusion, IL-17 is the key mediator of acute lung injury in AE-IPF, which might be secreted by Th17, γδT, pathologic Treg, and regenerating epithelial cells. The main effector cells of IL-17 are proliferating AECs and myofibroblasts (106).

PD-1 is a membrane molecule on T lymphocytes, with a major effect of inhibiting immune activity in chronic inflammation, such as malignant tumors, chronic infection, and IPF (107). It has been demonstrated that PD-1 cell surface expression was significantly higher on IPF CD4+ T cells compared to healthy controls. Peripheral PD-1+CD4+ T cells secreted more TGFβ and IL-17A in IPF compared with healthy controls (108). T lymphocytes from LLN of patients with ILD also highly expressed PD-1 (59). Mechanistically, PD-1 seems to be a pro-fibrotic molecule (108). In the steady phase of pulmonary fibrosis, it might aggravate fibrosis by inducing TGFβ secretion (54). If patients with pulmonary fibrosis have an acute infection, the inhibitory effect of PD-1 would cause uncontrolled acute inflammations. Because of the higher expression of PD-1, the adaptive immune reaction to infection is exhausted, exhibiting insufficient proliferation and differentiation of T lymphocytes and deficiency of antibodies (109). This will cause extra activation of innate immune reactivity with an explosive increase of neutrophil and monocyte infiltration, which eventually induces acute lung injuries (110). Further studies are needed to explore the roles of PD-1 and related T-cell exhaustion markers in AE-IPF.

In conclusion, in patients with stable IPF, CD4+ T lymphocytes are in the inhibitory status, with increased PD-1 and decreased CD28 and ICOS. Once there is an episode of acute infection, these CD4+ T lymphocytes produce IL-17 and initiate acute inflammations (111).

## B cells and plasma cells in AE-IPF

In patients with AE-IPF, there is a lack of evidence for the roles of B cells. However, autoantibody reduction therapy, including therapeutic plasma exchange, rituximab, and intravenous immunoglobulin, has benefited patients with AE-IPF (112). Therefore, a phase IIb clinical trial to determine the efficacy of autoantibody reduction therapy among patients with AE-IPF was ongoing (113).

In patients with IPF, the roles of humoral immunity remain to be illustrated. Previous studies on humoral immunity in IPF had opposing conclusions. Some studies found that patients with IPF had more circulating activated B lymphocytes and autoreactive IgA, which were associated with disease progression (114). These activated B cells stimulated differential fibroblast migration and activation in the lung tissues of patients with IPF (115). Moreover, a specific phenotype of B cell, expressing FCRL5, was present in patient with AE-IPF (69). Before activated B cells terminally differentiate into plasma cells, plasmablasts are an intermediate cell type that express both B cell (CD19+) and plasma cell (CD38+CD27+) surface markers. It has been demonstrated that the proportion of plasmablasts in B cells is increased in the peripheral blood of patients with IPF, compared with healthy individuals, and the B lymphocyte stimulator, also known as the B-cell activating factor (BAFF), is higher in the plasma of patients with IPF (116). In a recent study, Elisabetta and colleagues found that B cells were enriched in lung tissues of early- and end-stage IPF (117). However, other studies found that, at diagnosis, patients with IPF showed fewer B lymphocytes in peripheral blood (118). Additionally, the ratio of Breg cells to total B cells was significantly decreased in patients with IPF, which was positively correlated with a decline in pulmonary function (107). In another study, researchers performed a mass cytometry analysis of immune cell subsets in BALF from patients with IPF, CTD-ILD, and sarcoidosis, and they found that B cells were significantly decreased in IPF compared with CTD-ILD and sarcoidosis (69). Furthermore, in the mouse model of pulmonary fibrosis, deficiency in B cells did not reduce the lung tissue remodeling and worsened lung function (119), either by knockout μMT or by anti-CD20 B-cell ablation therapy. However, depleting plasma cells reduced the level of bleomycin-induced lung fibrosis in mice (120). Overall, the effect of B lymphocytes in different stages of IPF needs further delineation.

In summary, compared with healthy people, patients with IPF show increased B cells in peripheral blood and lung tissues, which are activated after exposure to autoantigens during lung tissue destruction. However, compared with patients with CTD-ILD, humoral immunity might be activated at a lower level, as B-cell activation and maturation partly depend on the antigen -presentation and T-helper cells, which are insufficient in IPF.

## The alterations of bone marrow in IPF

Bone marrow is involved in the development of pulmonary fibrosis and is especially important for acute exacerbations of IPF (**Figure 2**). In a previous study, researchers found that a low-dose bleomycin insult led to an alteration of bone marrow, which caused severe pulmonary fibrosis in recipient mice (84). Bone marrow is the source of myeloid immune cells. In patients with IPF, the attracted myeloid cells enhanced pulmonary fibrosis, and elimination of these cells reduced pulmonary fibrosis (121). A previous study found that a bone marrow transplant can prevent the progression of pulmonary fibrosis in a patient with refractory autoimmune diseases (122). The repetitive microinjuries of AECs can cause alterations in BM cells, but the altered BM, in turn, can also affect disease in distal organs (123). How the pulmonary immune environment communicates with bone marrow is another key point to clarify the immune mechanism of AE-IPF. Cytokines and chemokines are essential in this process, including CXCL1, CCL2, sB7H3, IL-17A, G-CSF, and GM-CSF. IL-17A is mainly secreted by lymphocytes, while sB7H3 is produced by antigen-presenting cells, like macrophages and dendritic cells. G-CSF, GM-CSF, CCL2, and CXCL1 are mainly produced by activated alveolar epithelium and endothelium (124). IL-17A can promote the secretion of these cytokines and chemokines by acting on activated alveolar epithelium and endothelium. Researchers have found that GM-CSF and G-CSF were elevated in BALF of IPF, which was correlated with increased BALF neutrophil and eosinophil count and disease progression of IPF (125, 126). Increased levels of G-CSF and GM-CSF were also observed in AE-IPF, which stimulated the proliferation of myeloid hematopoietic cells (39). Then, monocytes and neutrophils are recruited to lung tissues, with the help of chemokines. Subsequently, monocytes differentiate into Mo-M. CCL2, CCL7, and CCL13 are key chemokines for recruiting monocytes to inflammatory areas of lung tissues (127, 128). Knockout of CCL2 in mice led to milder lung fibrosis and less mononuclear phagocyte after bleomycin administration (129). Furthermore, CCL2 is also increased in BALF of patients with AE-IPF and is associated with worse outcomes of AE-IPF (130). CXCL1 is important for neutrophil accumulation in AE-IPF, as it is increased significantly in BALF of the AE-IPF mouse model (39, 131). Further research to depict the alteration of cell phenotype and molecular mechanisms in the bone marrow might help predict acute exacerbations and treatment in advance of AE-IPF. We believe that the above immune mechanisms also explain the immune disturbance of other chronic inflammatory conditions, including malignant tumors and autoimmune disorders (132). In malignant tumors, a subset of disease-specific macrophages is identified as TAMs, which are an M2 phenotype and exhibit inhibitory functions in inflammation and pro-tumor functions during the development of tumors (133). Moreover, the exhausted T lymphocytes and prominent Th17/Treg reaction in patients with tumor are quite similar to the immune reaction of patients with IPF (134, 135).

## Future research directions

First, previous studies indicated that tissue-resident memory T cells (TRM) were also involved in the fibrotic process. After being activated by antigen-presenting cells, a small proportion of naïve T cells differentiate into memory T cells and reside in lung tissues as TRM after the inflammation is resolved. By applying mass cytometry to quantify immune cell subsets in the lungs of patients with IPF, researchers demonstrated that CD4+ TRM and CD8+ TRM were increased in the lungs of IPF (136). However, there needs further investigation of the heterogeneity of TRM cell contributions to the pathobiology of IPF and AE-IPF.

Second, the over-activation of innate immunity is an essential event of AE-IPF. However, corticosteroids, a widely used immunosuppressive agent, mainly inhibited lymphocytes’ reaction and proliferation, but have little effect on innate immunity. That is why corticosteroids have poor treatment effects on AE-IPF, even with a high dose. Therefore, there is an urgent need to develop a novel treatment measure, targeting innate immunity. Furthermore, SAM is differentiated from monocytes, but not all Mo-M exhibit a profibrotic phenotype; some are helpful in alveolar epithelial repair (137). To date, there has not been a study that explores what determines the function of Mo-M. Future studies should focus on the molecules or pathways that determine whether macrophages function in pro-fibrosis or pro-repair. Furthermore, animal studies have demonstrated that a population of Mo-M persisted in the lung for 1 year and finally became similar to TR-AM (138). However, the fibrosis process is sustained in patients with IPF; thus, it is unclear whether SAM in IPF undergoes a cell death process like apoptosis or differentiation into TR-AM. Furthermore, as most lung tissues in IPF are undergoing a pro-fibrosis process, macrophages are generally in the M2 stage. In this condition, once the patients encounter other stimulations such as infection (bacteria or virus), injury (surgical operation), or aspiration (smoke or air pollution), the immune system depends on the M2 to cope with these stimuli, which is different from the normal situation with TR-AM (M0). It remains to be elucidated how the immune system of patients with IPF reacts to infections and other stimuli, which is the fundamental mechanism of AE-IPF.

Third, the differences between patients with IPF and animal models prevent the translation of mechanistic studies. Although the bleomycin-induced mouse model has been recognized as the best-characterized model of lung fibrosis with highly relevant pathobiology, there are also some limitations. For example, as we mentioned before, mechanical injuries might be an important factor initiating the AE-IPF, the central distribution of fibrotic lesions in the bleomycin model cannot catch the biomechanical properties of patients with IPF, which is subpleural distribution. Thus, a murine model that better simulates the clinical situation might enable us to deeply explore the biomechanical changes in AE-IPF.

Last, except for the immune cells, fibroblasts and vascular endothelial cells are also involved in the immune reactions of AE-IPF. For example, fibroblasts have been classified into alveolar and adventitial fibroblasts based on anatomical location in the respiratory tract. Functionally, alveolar fibroblasts can restrict inflammation, and loss of this niche abrogates fibrosis but exacerbates lung inflammation (139). However, further exploration is needed for a detailed understanding of basic fibroblast biology in the development of AE-IPF.

## Conclusions

In patients with IPF, the immune balance of lung tissues and bone marrow is disturbed, which weakens the ability to cope with external stimuli, including infections and aspirations. In the fibrotic lung tissues, macrophages are transformed into a specific phenotype, which is deficient in antigen recognition, phagocytosis, and antigen presentation. Lymphocytes are inhibited with decreased proliferation rates and differentiative potential due to decreased expression of costimulatory molecules (CD28 and ICOS). In patients with AE-IPF, injured AECs release GM-CSF and G-CSF to initiate acute inflammation. With the help of chemokines, neutrophils and Mo-M are engaged in the process of acute lung injury by secreting acute inflammatory cytokines (IL-6, IL-23, and TNFα) (140), which transform innate lymphocytes and Treg into Th17. Th17 secretes IL-17A and IL-22 acting on AECs to promote releasing GM-CSF and G-CSF. The positive feedback loop eventually leads to acute exacerbations of IPF. However, trying to intervene at a certain stage of this cycle fails to achieve clinical efficacy. Therefore, there is an urgent need to further explore the mechanisms so as to develop individualized treatment.
